# Estimating Risks and Relative Risks in Case-Base Studies under the Assumptions of Gene-Environment Independence and Hardy-Weinberg Equilibrium

**DOI:** 10.1371/journal.pone.0105398

**Published:** 2014-08-19

**Authors:** Tina Tsz-Ting Chui, Wen-Chung Lee

**Affiliations:** 1 Institute of Epidemiology and Preventive Medicine, College of Public Health, National Taiwan University, Taipei, Taiwan; 2 Research Center for Genes, Environment and Human Health, College of Public Health, National Taiwan University, Taipei, Taiwan; Sanjay Gandhi Medical Institute, India

## Abstract

Many diseases result from the interactions between genes and the environment. An efficient method has been proposed for a case-control study to estimate the genetic and environmental main effects and their interactions, which exploits the assumptions of gene-environment independence and Hardy-Weinberg equilibrium. To estimate the absolute and relative risks, one needs to resort to an alternative design: the case-base study. In this paper, the authors show how to analyze a case-base study under the above dual assumptions. This approach is based on a conditional logistic regression of case-counterfactual controls matched data. It can be easily fitted with readily available statistical packages. When the dual assumptions are met, the method is approximately unbiased and has adequate coverage probabilities for confidence intervals. It also results in smaller variances and shorter confidence intervals as compared with a previous method for a case-base study which imposes neither assumption.

## Introduction

Many diseases result from the interactions between genes and the environment [1], [2]. A case-control study will provide estimates of the genetic and environmental main effects (in terms of odds ratios), and their interactions (in terms of ratios of odds ratios). It is often reasonable to assume that among the non-diseased subjects in the study population, the gene under study is in Hardy-Weinberg equilibrium (which will be achieved within one generation of random mating in any population [3]) and is independent of exposures (genes being constitutional and environmental exposures being exogenous, are often uncorrelated to each other). Previous studies have demonstrated that imposing the dual assumptions of gene-environment independence and Hardy-Weinberg equilibrium can greatly improve the statistical efficiency of a case-control study [4]–[8].

However, in addition to odds ratios, we may also be interested in knowing the relative and absolute risks of subjects with different genetic and environmental profiles in the population. The relative risk is the ratio of the disease risk for individuals with one specific genetic and environmental profile, to the disease risk for those at a reference level. While indices of relative risk and odds ratio are equally suitable for etiologic inferences, a relative risk (a ratio of two risks) is easier to follow than an odds ratio (a ratio of two ‘odds’; but what is an odds?). The risk itself (or the absolute risk, to be precise) is also important; it is the disease probability for an individual with a specific genetic and environmental profile, and should be a clinically valuable index. Unfortunately, a case-control design does not provide estimates for the absolute risks; without a rare-disease assumption, estimates for the relative risks are also not provided.

A case-base design is an attractive alternative to the case-control design [9]–[13]. In contrast to the case-control study which samples the non-diseased subjects in the study base as the control group, the case-base study samples the entire study base without regard to disease status. The design directly produces a relative risk estimate without resorting to the rare-disease assumption [9]–[12]. Recently, Chui and Lee [13] described a logistic model for case-base study which can be easily fitted using existing statistical software to produce odds ratio estimates and, upon one additional step of simple calculations of the model parameters, relative and absolute risk estimates as well. However, Chui and Lee [13] did not elaborate on how to incorporate the assumptions of gene-environment independence and Hardy-Weinberg equilibrium into the model to further improve statistical efficiency.

In this paper, we show how to analyze case-base study assuming the above dual assumptions. We perform a Monte-Carlo simulation to investigate the statistical performance of the proposed method.

## Methods

### Case-Base Study Assuming Gene-Environment Independence and Hardy-Weinberg Equilibrium

Let 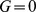, 1 and 2 represent the number of the variant allele(s) a subject carries. We define two dummy variables: 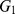 and 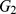, with 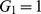, if 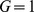, and 0 if otherwise; 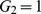, if 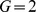, and 0 if otherwise. Let 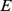 be the exposure status of a subject which can be in any measurement scale: binary, ordinal or continuous. Let 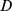 represent the disease status of a subject, with 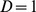 for diseased and 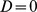 for non-diseased. We assume that the disease risk in the study population follows a logistic model:
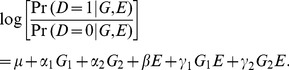
(1)


The 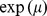 is the baseline disease odds in the population; 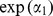 is the odds ratio of disease for those with 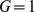 vs. those with 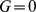; and 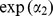, the odds ratio for those with 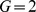 vs. those with 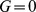. The 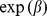 is the odds ratio associated with the environmental exposure. The 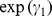 and 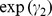 are the odds ratios associated with gene-environment interactions.

In a case-base study, researchers implement two sampling schemes: the ‘case’ and the ‘control’ sampling schemes [9]-[13]. The case sampling scheme targets the diseased subjects. Let 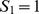 indicate that a diseased subject is recruited in the case sample of a case-base study; 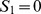, otherwise. In such a case sampling scheme:
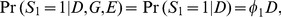
(2)where 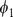 is a constant between 0 and 1. Note that the sampling probability depends only on 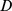, not on 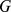 and 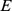. In this sampling scheme, the diseased subjects have a constant non-zero probability of being recruited [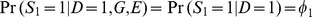], whereas the non-diseased subjects have a probability of zero of being recruited [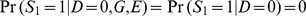].

The control sampling scheme targets the entire population (the study base) without regard to disease status. Let 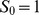 indicate that a subject is recruited in the control sample; 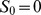, otherwise. Such a control sampling scheme is noted as:
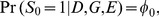
(3)where 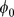 is a constant between 0 and 1. Note that this sampling scheme essentially is a random sampling of the study population at large; it depends on neither 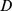, 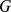 nor 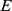.

The two sampling schemes are assumed to be independent of each other. A diseased subject can be recruited in a case-base study simultaneously in the case sample and in the control sample. The probability of a diseased subject entering the case-base study as a duplicate sample (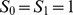) is:
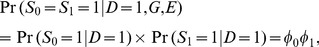
(4)which depends on neither 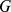 nor 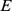. The event of 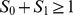 indicates that a subject is recruited in a case-base study through case sampling, control sampling or both. Let 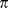 be the probability that a diseased subject recruited in a case-base study can be found in the control sample, i.e.:
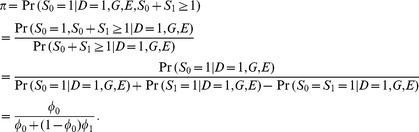
(5)


Again, this depends on neither 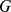 nor 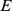.

The maximum likelihood estimate of 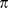 and its variance (see Chui and Lee [13]) are:
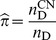
(6)and
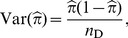
(7)where 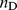 is the total number of *distinct* diseased subject recruited in the case-base study, and 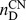 is the number of diseased subjects recruited in the control sample. Chui and Lee [13] showed that the disease risk in a case-base sample also follows a logistic model:
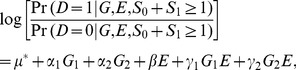
(8)where 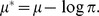


As in Lee et al. [8], we assume that among the non-diseased subjects in the study population, the gene (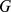) is independent of environmental exposure (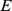) [the first equality in the following Equation (9)] and in the Hardy-Weinberg equilibrium (the second equality), i.e.:
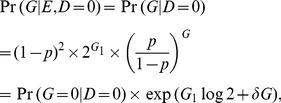
(9)where 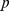 is the allele frequency and 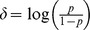, the log allele frequency odds, among the non-diseased subjects in the study population. Combining Equations (8) and (9), the likelihood function for a case-base study under the assumptions of gene-environment independence and Hardy-Weinberg equilibrium is found to be (Exhibit S1):
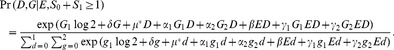
(10)


Model (10) above has exactly the same form of the likelihood function of a 1∶5 matched case-control study. [The denominator in Model (10) has a total of six terms, corresponding to one ‘case’ and five ‘controls’.] Therefore, one can adopt the ‘counterfactual approach’ [8] to fit Model (10). To be precise, one first creates a recruitment indicator, 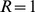, for each and every distinct subject actually recruited in the case-base study. Next, one creates a total of five counterfactual subjects (all of them with 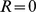) to each recruited subject; the exposure status (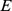) of the five counterfactual subjects is deliberately set to be exactly the same as the recruited subject to whom they are matched, but the disease status (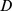) and gene (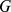) are different. (The five subjects represent the five different ways of making [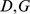]-different counterfactuals.) Treating 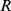 as the outcome variable, one then performs a conditional logistic regression analysis (using existing statistical software, such as SAS) with the following regression equation: 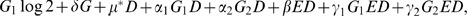 based on the above created 1 (factual): 5 (counterfactuals) matched data. The results are the conditional maximum likelihood estimates of the total 7 parameters in Model (10), together with their variance-covariance matrix, 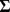, a 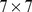 matrix.

Among the parameter estimates obtained from a fitting of Model (10) to data, 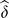 is an estimate for log allele frequency odds in Equation (9), 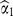 and 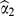 are estimates for log genetic odds ratios in Model (1), 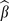 is an estimate for log environmental odds ratio in Model (1), and 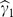 and 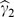 are estimates associated with gene-environment interactions in Model (1). The 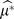 estimate from Model (10) is to be further combined with the 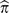 estimate from Equation (6) to provide an estimate for the log baseline disease odds in Model (1):



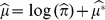
(11).

Because 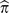 is independent of 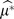 [13], the variance of 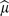 is:
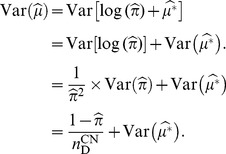
(12)


Now, we can estimate the absolute and relative risks. An estimate of the (absolute) disease risk for subjects in the study population with (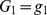,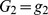,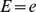) is:
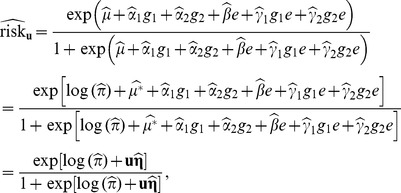
(13)


where 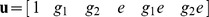 is a 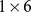 gene-environment profile vector and 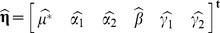 is a 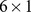 vector of parameter estimates. Because 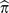 is independent of all the other parameters [13], the variance of the estimate (in logit scale) is:
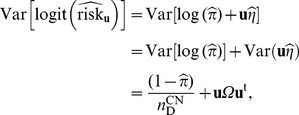
(14)where 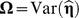 is readily available by simply deleting the first row and the first column of 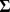. [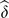 from Model (10) plays no role in the disease risk estimation.] Let the reference group be those subjects in the study population with (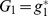,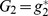,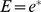), and let 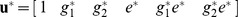 be the gene-environment profile vector for them. An estimate of the relative risk is therefore:
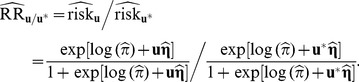
(15)


Using the delta method, the variance of the estimate (in log scale) is:
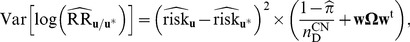
(16)where 
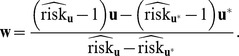


### Monte-Carlo Simulations

For simplicity, we assume a binary exposure *E* (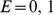) and a biallelic gene with genotype *G* (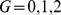). For the non-diseased subjects in the study population, we assume gene-environment independence and Hardy-Weinberg equilibrium, with the exposure prevalence (for 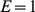) and the allele frequency of the variant allele both being set at 0.5. The disease probabilities of subjects in the study population are assumed to follow the logistic model [Model (1)]. We assume an autosomal recessive gene with a genetic odds ratio of 2.0 [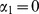 and 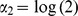], an environmental odds ratio of 2.5 [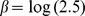] and a gene-environmental interaction odds ratio of 2.0 [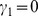 and 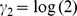]. The disease prevalence in the study population is set at 0.1. Therefore, the six disease risks are: 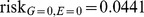, 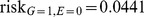, 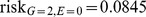, 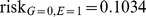, 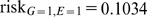, and 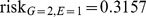, respectively. And the five relative risks are (with 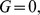 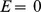 as the reference level) 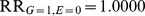, 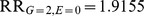, 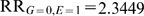, 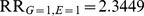, and 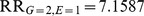, respectively. A case-base study is conducted in the study population (population size: 100,000) with a case sampling probability (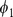) of 0.05 and a control sampling probability (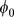) of 0.005. Under such a sampling scheme, the case-base study is expected to recruit a total of 500 distinct diseased and 500 distinct non-diseased subjects. Exhibit S2 presents the SAS code for simulating data.

Using the formulas derived by Cheng and Lin [6], we compare the relative efficiencies of the estimates in the conditional logistic regression [with the assumptions of gene-environment independence and Hardy-Weinberg equilibrium, Model (10)] relative to the corresponding estimates in Chui and Lee's [13] unconditional logistic regression [without the dual assumptions, Model (8)]. [Relative efficiency of A method relative to B method is defined as the ratio of the variance of B estimator and that of A estimator. A larger-than-one relative efficiency implies a better statistical performance (larger power and better precision, etc) for A method as compared to B method.]

In regard to the estimates for relative risks and absolute risks, we perform a total of 10,000 simulations to compare the performances of the two approaches (the conditional logistic regression with the dual assumptions vs. the unconditional logistic regression without the dual assumptions). The means of the estimates for relative risks (in log scale) and absolute risks (in logit scale) are calculated. The variance of an estimate is calculated as the sample variance of the estimates. We also calculate the coverage probabilities and the average lengths of the 95% confidence intervals for the estimates. [The coverage probability of a 95% confidence interval for a parameter estimate is the probability that the interval covers the true value of the parameter in a repeated sampling (simulation) experiment.]

## Results

The relative efficiency of the proposed method (with the dual assumptions of gene-environment independence and Hardy-Weinberg equilibrium), as compared to Chui and Lee's method (without the dual assumptions) [13], is shown in Exhibit S3. It can be seen that exploiting the dual assumptions can greatly improve statistical efficiency. The relative efficiencies are 1.30 (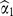), 1.44 (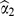), 1.37 (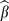), 1.57 (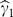), and 1.85 (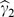), respectively.

Table 1 shows the simulation results for the estimates for relative and absolute risks. Using either approach, the estimates of relative and absolute risks are approximately unbiased. [The bias of a parameter estimate is the difference between the mean of the parameter estimates in the simulation experiment and the true value of that parameter; compare the column labeled ‘Estimate’ and the column labeled ‘True value’ in Table 1.] The 95% confidence intervals also achieve adequate coverage probabilities for both approaches. However, the variances and the average lengths of the confidence intervals using the present method, which imposes the dual assumptions of gene-environment independence and Hardy-Weinberg equilibrium, are much smaller than Chui and Lee's method [13] which imposes neither assumption.

**Table 1:** Simulation results for a biallelic gene and a binary exposure with and without the assumptions of gene-environment independence and Hardy-Weinberg equilibrium.

Log relative risk or logit absolute risk		Estimate		Variance		Coverage probability of 95% confidence interval		Average length of 95% confidence interval	
	True value	with assumptions	without assumptions	with assumptions	without assumptions	with assumptions	without assumptions	with assumptions	without assumptions
logRR*_G_*_ = 1, *E* = 0_	0.0000	0.0135	0.0069	0.0533	0.0725	0.9534	0.9535	0.8980	1.0537
logRR*_G_*_ = 2, *E* = 0_	0.6500	0.6632	0.6579	0.0616	0.0707	0.9506	0.9495	0.9608	1.0275
logRR*_G_*_ = 0, *E* = 1_	0.8522	0.8524	0.8602	0.0830	0.1369	0.9540	0.9513	1.1204	1.4368
logRR*_G_*_ = 1, *E* = 1_	0.8522	0.8663	0.8629	0.0621	0.0807	0.9533	0.9518	0.9740	1.1097
logRR*_G_*_ = 2, *E* = 1_	1.9683	1.9846	1.9810	0.0611	0.0693	0.9513	0.9517	0.9619	1.0288
logit(risk*_G_*_ = 0,*E* = 0_)	−3.0762	−3.1022	−3.0980	0.0695	0.0793	0.9530	0.9531	1.0258	1.0984
logit(risk*_G_*_ = 1,*E* = 0_)	−3.0762	−3.0888	−3.0914	0.0348	0.0379	0.9495	0.9500	0.7284	0.7608
logit(risk*_G_*_ = 2,*E* = 0_)	−2.3831	−2.3959	−2.3934	0.0330	0.0355	0.9485	0.9484	0.7096	0.7351
logit(risk*_G_*_ = 0,*E* = 1_)	−2.1599	−2.1850	−2.1707	0.0807	0.1241	0.9515	0.9501	1.0914	1.3479
logit(risk*_G_*_ = 1,*E* = 1_)	−2.1599	−2.1714	−2.1720	0.0411	0.0507	0.9542	0.9520	0.7937	0.8772
logit(risk*_G_*_ = 2,*E* = 1_)	−0.7736	−0.7828	−0.7765	0.0343	0.0455	0.9506	0.9552	0.7258	0.8414

## Discussion

Lee et al. [8] previously discussed how to relax the Hardy-Weinberg equilibrium for case-control studies. This can also be applied to the present context of case-base studies. Assuming only the gene-environment independence assumption, the case-base likelihood becomes:
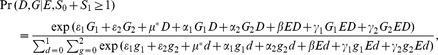
where 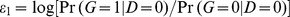 and 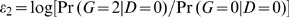 are gene-frequency-related parameters (log genotype frequency odds among the non-diseased subjects in the study population, to be precise). By comparison, the likelihood function (Model 10) where both assumptions are imposed contains only one gene-frequency related parameter (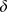).

If the study population is not homogeneous, but is instead composed of a number of population strata, a case-base study is also vulnerable to population stratification biases just as a case-control study can be. Assume that there are a total of 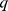 stratum; the case-base likelihood conditioned on stratum indicators, 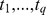, is:
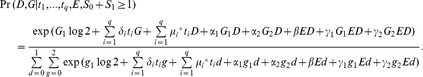



The interaction terms 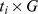 (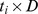) allows the allele frequency odds (the background disease odds) to vary between different population strata. To use this model, one needs to know in advance the stratum to which each and every study subject belongs.

In this paper, we present a method to analyze the case-base study exploiting the assumptions of gene-environment independence and Hardy-Weinberg equilibrium with common statistical packages. When both assumptions are met, the simulation results show that the method is approximately unbiased and has adequate coverage probabilities of the 95% confidence intervals. It also results in smaller variances and shorter confidence intervals as compared to a previous assumption-free method for a case-base study.

## Supporting Information

Exhibit S1 **Derivation of the likelihood function for a case-base study under the assumptions of gene-environment independence and Hardy-Weinberg equilibrium.**
(PDF)

Exhibit S2 **SAS code for simulating data.**
(PDF)

Exhibit S3 **Calculation of the relative efficiency of the proposed conditional logistic regression method (with the dual assumptions of gene-environment independence and Hardy-Weinberg equilibrium) as compared to the unconditional logistic regression method (without the dual assumptions).**
(PDF)
