# Supplementary material for: Estimating Risks and Relative Risks in Case-Base Studies under the Assumptions of Gene-Environment Independence and Hardy-Weinberg Equilibrium
Source: PLoS One. 2014 Aug 19;9(8):e105398. doi: 10.1371/journal.pone.0105398 (PMC4138174; doi:10.1371/journal.pone.0105398)
Supplement: Exhibit S1 — Derivation of the likelihood function for a case-base study under the assumptions of gene-environment independence and Hardy-Weinberg equilibrium. (PDF) [file pone.0105398.s001.pdf]

Exhibit S1. Derivation of the likelihood function for a case-base study under the assumptions of gene-environment independence and Hardy-Weinberg equilibrium.

In a case-base study, the recruitment process depends on a subject's disease status but not on his/her genotype and exposure status, that is,

$$\Pr\left(S_0 + S_1 \geq 1 \mid D, G, E\right) = \Pr\left(S_0 + S_1 \geq 1 \mid D, E\right) = \Pr\left(S_0 + S_1 \geq 1 \mid D\right).$$

From Equation (9) in text, we have,

$$\begin{aligned}
&\Pr(D = 0, G \mid E, S_0 + S_1 \geq 1) \\
&\quad = \Pr(D = 0 \mid E, S_0 + S_1 \geq 1) \times \Pr(G \mid D = 0, E, S_0 + S_1 \geq 1) \\
&\quad = \Pr(D = 0 \mid E, S_0 + S_1 \geq 1) \times \frac{\Pr(S_0 + S_1 \geq 1 \mid D = 0, G, E) \times \Pr(G \mid D = 0, E) \times \Pr(D = 0, E)}{\Pr(S_0 + S_1 \geq 1 \mid D = 0, E) \times \Pr(D = 0, E)} \\
&\quad = \Pr(D = 0 \mid E, S_0 + S_1 \geq 1) \times \Pr(G \mid D = 0, E) \\
&\quad = \Pr(D = 0 \mid E, S_0 + S_1 \geq 1) \times \Pr(G = 0 \mid D = 0) \times \exp(G_1 \log 2 + \delta G).
\end{aligned}$$

From Model (8) in text, we have

$$\begin{aligned}
&\Pr(D = 1, G \mid E, S_0 + S_1 \geq 1) \\
&\quad = \Pr(D = 0, G \mid E, S_0 + S_1 \geq 1) \times \exp(\mu^* + \alpha_1 G_1 + \alpha_2 G_2 + \beta E + \gamma_1 G_1 E + \gamma_2 G_2 E),
\end{aligned}$$

and therefore,

$$\begin{aligned}
\Pr(D = 1, G \mid E, S_0 + S_1 \geq 1) &= \Pr(D = 0 \mid E, S_0 + S_1 \geq 1) \times \Pr(G = 0 \mid D = 0) \\
&\quad \times \exp(G_1 \log 2 + \delta G + \mu^* + \alpha_1 G_1 + \alpha_2 G_2 + \beta E + \gamma_1 G_1 E + \gamma_2 G_2 E).
\end{aligned}$$

We see that both $\Pr\left(D = 1, G \mid E, S_0 + S_1 \geq 1\right)$ and $\Pr\left(D = 0, G \mid E, S_0 + S_1 \geq 1\right)$ have the same multiplier: $\Pr\left(D = 0 \mid E, S_0 + S_1 \geq 1\right) \times \Pr\left(G = 0 \mid D = 0\right)$. It immediately follows that the

likelihood function for a case-base study under the dual assumptions of gene-environment independence and Hardy-Weinberg equilibrium is

$$\Pr(D, G \mid E, S_0 + S_1 \geq 1)$$
$$= \frac{\exp(G_1 \log 2 + \delta G + \mu^* D + \alpha_1 G_1 D + \alpha_2 G_2 D + \beta ED + \gamma_1 G_1 ED + \gamma_2 G_2 ED)}{\sum_{d=0}^{1} \sum_{g=0}^{2} \exp(g_1 \log 2 + \delta g + \mu^* d + \alpha_1 g_1 d + \alpha_2 g_2 d + \beta Ed + \gamma_1 g_1 Ed + \gamma_2 g_2 Ed)}.$$
