# Supplementary material for: Estimating Risks and Relative Risks in Case-Base Studies under the Assumptions of Gene-Environment Independence and Hardy-Weinberg Equilibrium
Source: PLoS One. 2014 Aug 19;9(8):e105398. doi: 10.1371/journal.pone.0105398 (PMC4138174; doi:10.1371/journal.pone.0105398)
Supplement: Exhibit S2 — SAS code for simulating data. (PDF) [file pone.0105398.s002.pdf]

Exhibit S2. SAS code for simulating data.

```
DM LOG 'clear'; DM OUTPUT 'clear';
options linesize=80;

%let    pe0 = 0.5;
%let    pe1 = 0.5;
%let    pa=0.5;

%let mu=log(0.0441/0.9559);
%let alpha1 = log(1);
%let alpha2 = log(2);
%let beta = log(2.5);
%let gamma1 = log(1);
%let gamma2 = log(2);

%let D1G0E0 = exp(&mu);                                        %let D0G0E0 = exp(0);
%let D1G1E0 = exp(log(2)+ (&pa/(1-&pa))+ &mu+ &alpha1);    %let D0G1E0 = exp(log(2)+ (&pa/(1-&pa)));
%let D1G2E0 = exp(2*(&pa/(1-&pa))+ &mu+ &alpha2);          %let D0G2E0 = exp(2*(&pa/(1-&pa)));

%let D1G0E1 = exp(&mu+ &beta);                                        %let D0G0E1 = exp(0);
%let D1G1E1 = exp(log(2)+ (&pa/(1-&pa))+ &mu+ &alpha1+ &beta+ &gamma1);    %let D0G1E1 = exp(log(2)+ (&pa/(1-&pa)));
%let D1G2E1 = exp(2*(&pa/(1-&pa))+ &mu+ &alpha2+ &beta+ &gamma2);          %let D0G2E1 = exp(2*(&pa/(1-&pa)));
```

```
%let baseE0 = (&D1G0E0)+(&D1G1E0)+(&D1G2E0)+(&D0G0E0)+(&D0G1E0)+(&D0G2E0);
%let baseE1 = (&D1G0E1)+(&D1G1E1)+(&D1G2E1)+(&D0G0E1)+(&D0G1E1)+(&D0G2E1);

%let pd00 = (&D1G0E0) / (&baseE0);   %let pb00 = (&D0G0E0) / (&baseE0);
%let pd10 = (&D1G1E0) / (&baseE0);   %let pb10 = (&D0G1E0) / (&baseE0);
%let pd20 = (&D1G2E0) / (&baseE0);   %let pb20 = (&D0G2E0) / (&baseE0);
%let pd01 = (&D1G0E1) / (&baseE1);   %let pb01 = (&D0G0E1) / (&baseE1);
%let pd11 = (&D1G1E1) / (&baseE1);   %let pb11 = (&D0G1E1) / (&baseE1);
%let pd21 = (&D1G2E1) / (&baseE1);   %let pb21 = (&D0G2E1) / (&baseE1);

%let          mm = 500;
%let          nn     = 500;
%let          nsim= 1; /**** number of simulations *****/

FILENAME OUT DUMMY '/DEV/NULL';
PROC PRINTTO LOG=OUT;

%macro ccsim (nsim, pe0, pe1, nn, mm);
   %do i = 1 %to &nsim;

        data sample;
              drop x y z1 z2 z3 z4 w1 w2 w3 w4;
              n=0;m=0; ID=0;
              do while (n < &nn);
              x=rand('uniform');
```

```
        y=rand('uniform');
            z1=(&pd00) + (&pb00);   z2=(&pd00) + (&pb00) + (&pd10);   z3=(&pd00) + (&pb00) + (&pd10) + (&pb10);
            z4=(&pd00) + (&pb00) + (&pd10) + (&pb10) + (&pd20);
            w1=(&pd01) + (&pb01);   w2=(&pd01) + (&pb01) + (&pd11);   w3=(&pd01) + (&pb01) + (&pd11) + (&pb11);
            w4=(&pd01) + (&pb01) + (&pd11) + (&pb11) + (&pd21);

            if x <= (&pe0) then E=0;
                else E=1;

        if E=0 & y <= (&pd00) then do; D=1; G=0; G1=0; G2=0; S0=1; S1=0; m=m+1; ID=ID+1; output; end;
    else if E=0 & y <= (z1) then do; D=0; G=0; G1=0; G2=0; S0=1; S1=0; n=n+1; ID=ID+1;output; end;
    else if E=0 & y <= (z2) then do; D=1; G=1; G1=1; G2=0; S0=1; S1=0; m=m+1;   ID=ID+1; output; end;
    else if E=0 & y <= (z3) then do; D=0; G=1; G1=1; G2=0; S0=1; S1=0; n=n+1; ID=ID+1; output; end;
    else if E=0 & y <= (z4) then do; D=1; G=2; G1=0; G2=1; S0=1; S1=0; m=m+1; ID=ID+1; output; end;
    else if E=0 & y > (z4) then do; D=0; G=2; G1=0; G2=1; S0=1; S1=0; n=n+1; ID=ID+1; output; end;

else    if E=1 & y <= (&pd01) then do; D=1; G=0; G1=0; G2=0; S0=1; S1=0; m=m+1; ID=ID+1; output; end;
    else if E=1 & y <= (w1) then do; D=0; G=0; G1=0; G2=0; S0=1; S1=0; n=n+1; ID=ID+1; output; end;
    else if E=1 & y <= (w2) then do; D=1; G=1; G1=1; G2=0; S0=1; S1=0; m=m+1; ID=ID+1; output; end;
    else if E=1 & y <= (w3) then do; D=0; G=1; G1=1; G2=0; S0=1; S1=0; n=n+1; ID=ID+1; output; end;
    else if E=1 & y <= (w4) then do; D=1; G=2; G1=0; G2=1; S0=1; S1=0; m=m+1; ID=ID+1; output; end;
    else if E=1 & y > (w4) then do; D=0; G=2; G1=0; G2=1; S0=1; S1=0; n=n+1; ID=ID+1; output; end;
        end;

        do while (m < &mm);
```

```
x=rand('uniform');
y=rand('uniform');
    if x <= (&pe0) then E=0;
        else E=1;

    if E=0 & y <= (&pd00) then do; D=1; G=0; G1=0; G2=0; S0=0; S1=1; m=m+1; ID=ID+1; output; end;
  else if E=0 & (z1) < y <= (z2) then do; D=1; G=1; G1=1; G2=0; S0=0; S1=1; m=m+1; ID=ID+1; output; end;
  else if E=0 & (z3) < y <= (z4) then do; D=1; G=2; G1=0; G2=1; S0=0; S1=1; m=m+1; ID=ID+1; output; end;
  else  if E=1 & y <= (&pd01) then do; D=1; G=0; G1=0; G2=0; S0=0; S1=1; m=m+1; ID=ID+1; output; end;
  else if E=1 & (w1) < y <= (w2) then do; D=1; G=1; G1=1; G2=0; S0=0; S1=1; m=m+1; ID=ID+1; output; end;
  else if E=1 & (w3) < y <= (w4) then do; D=1; G=2; G1=0; G2=1; S0=0; S1=1; m=m+1; ID=ID+1; output; end;
    end;
run;

proc freq data=sample;
    tables E*D/list;
    tables E*D*S0*S1/list;
tables D*S0/list;
run;

%end;
%mend;

%ccsim (&nsim, &pe0, &pe1, &nn, &mm);
```

```
FILENAME OUT DUMMY '/DEV/NULL';

PROC PRINTTO LOG=LOG; run;
```
