# Supplementary material for: Estimating Risks and Relative Risks in Case-Base Studies under the Assumptions of Gene-Environment Independence and Hardy-Weinberg Equilibrium
Source: PLoS One. 2014 Aug 19;9(8):e105398. doi: 10.1371/journal.pone.0105398 (PMC4138174; doi:10.1371/journal.pone.0105398)
Supplement: Exhibit S3 — Calculation of the relative efficiency of the proposed conditional logistic regression method (with the dual assumptions of gene-environment independence and Hardy-Weinberg equilibrium) as compared to the unconditional logistic regression method (without the dual assumptions). (PDF) [file pone.0105398.s003.pdf]

Exhibit S3. Calculation of the relative efficiency of the proposed conditional logistic regression method (with the dual assumptions of gene-environment independence and Hardy-Weinberg equilibrium) as compared to the unconditional logistic regression method (without the dual assumptions).

Under the simulation setup as described in text, the expected numbers of distinct subjects recruited in the case-base study are listed below:

	Disease Subjects		Non-Disease Subjects	
	$E=0$	$E=1$	$E=0$	$E=1$
$G=0$	26.2	61.4	66.8	62.6
$G=1$	52.3	122.7	133.6	125.3
$G=2$	50.1	187.3	64.0	47.8

The total numbers of non-diseased subjects are

$$62.6+125.3+47.8=235.7$$

for those with $E=1$, and

$$500-235.7=264.3$$

for those with $E = 0$, respectively.

The total allele counts among the non-diseased subjects are

$$133.6 + 125.3 + 2 \times 64.0 + 2 \times 47.8 = 482.3$$

for the variant allele, and

$$2 \times 500 - 482.3 = 517.7$$

for the wild-type allele, respectively.

The variances for the coefficient estimates in the conditional logistic regression method are

$$\mathrm{Var}\left(\hat{\alpha}_1^{\text{conditional}}\right) = \frac{1}{26.2} + \frac{1}{52.3} + \frac{1}{517.7} + \frac{1}{482.3} = 0.0613,$$
$$\mathrm{Var}\left(\hat{\alpha}_2^{\text{conditional}}\right) = \frac{1}{26.2} + \frac{1}{50.1} + \frac{4}{517.7} + \frac{4}{482.3} = 0.0742,$$
$$\mathrm{Var}\left(\hat{\beta}^{\text{conditional}}\right) = \frac{1}{26.2} + \frac{1}{61.4} + \frac{1}{264.3} + \frac{1}{235.7} = 0.0625,$$
$$\mathrm{Var}\left(\hat{\gamma}_1^{\text{conditional}}\right) = \frac{1}{26.2} + \frac{1}{52.3} + \frac{1}{61.4} + \frac{1}{122.7} = 0.0818,$$

and

$$\mathrm{Var}\left(\hat{\gamma}_2^{\text{conditional}}\right) = \frac{1}{26.2} + \frac{1}{50.1} + \frac{1}{61.4} + \frac{1}{187.3} = 0.0798,$$

respectively.

The variances for the coefficient estimates in the unconditional logistic regression method are

$$\mathrm{Var}\left(\hat{\alpha}_1^{\text{unconditional}}\right) = \frac{1}{26.2} + \frac{1}{52.3} + \frac{1}{66.8} + \frac{1}{133.6} = 0.0798,$$
$$\mathrm{Var}\left(\hat{\alpha}_2^{\text{unconditional}}\right) = \frac{1}{26.2} + \frac{1}{50.1} + \frac{1}{66.8} + \frac{1}{64.0} = 0.0888,$$

$$\text{Var}\left(\hat{\beta}^{\text{unconditional}}\right) = \frac{1}{26.2} + \frac{1}{61.4} + \frac{1}{66.8} + \frac{1}{62.6} = 0.0855,$$

$$\text{Var}\left(\hat{\gamma}_1^{\text{unconditional}}\right) = \text{Var}\left(\hat{\alpha}_1^{\text{unconditional}}\right) + \frac{1}{61.4} + \frac{1}{122.7} + \frac{1}{62.6} + \frac{1}{125.3}$$
$$= 0.1282,$$

and

$$\text{Var}\left(\hat{\gamma}_2^{\text{unconditional}}\right) = \text{Var}\left(\hat{\alpha}_2^{\text{unconditional}}\right) + \frac{1}{61.4} + \frac{1}{187.3} + \frac{1}{62.6} + \frac{1}{47.8}$$
$$= 0.1473,$$

respectively.

The relative efficiencies (RFs) are therefore,

$$\text{RF}\left(\hat{\alpha}_1\right) = \frac{\text{Var}\left(\hat{\alpha}_1^{\text{unconditional}}\right)}{\text{Var}\left(\hat{\alpha}_1^{\text{conditional}}\right)} = 1.30,$$

$$\text{RF}\left(\hat{\alpha}_2\right) = \frac{\text{Var}\left(\hat{\alpha}_2^{\text{unconditional}}\right)}{\text{Var}\left(\hat{\alpha}_2^{\text{conditional}}\right)} = 1.44,$$

$$\text{RF}\left(\hat{\beta}\right) = \frac{\text{Var}\left(\hat{\beta}^{\text{unconditional}}\right)}{\text{Var}\left(\hat{\beta}^{\text{conditional}}\right)} = 1.37,$$

$$\text{RF}\left(\hat{\gamma}_1\right) = \frac{\text{Var}\left(\hat{\gamma}_1^{\text{unconditional}}\right)}{\text{Var}\left(\hat{\gamma}_1^{\text{conditional}}\right)} = 1.57,$$

and

$$\text{RF}\left(\hat{\gamma}_2\right) = \frac{\text{Var}\left(\hat{\gamma}_2^{\text{unconditional}}\right)}{\text{Var}\left(\hat{\gamma}_2^{\text{conditional}}\right)} = 1.85,$$

respectively.
